# Low or High-Level Motor Coding? The Role of Stimulus Complexity

**DOI:** 10.3389/fnhum.2019.00332

**Published:** 2019-10-11

**Authors:** Lucia Amoruso, Alessandra Finisguerra

**Affiliations:** ^1^Basque Center on Cognition, Brain and Language, San Sebastian, Spain; ^2^IKERBASQUE, Basque Foundation for Science, Bilbao, Spain; ^3^Scientific Institute, IRCCS E. Medea, Udine, Italy

**Keywords:** action observation, motor resonance, kinematics mapping, top down modulations, motor evoked potentials, corticospinal excitability, transcranial magnetic stimulation

## Abstract

Transcranial magnetic stimulation (TMS) studies have shown that observing an action induces activity in the onlooker's motor system. In light of the muscle specificity and time-locked mirroring nature of the effect, this motor resonance has been traditionally viewed as an inner automatic replica of the observed movement. Notably, studies highlighting this aspect have classically considered movement in isolation (i.e., using non-realistic stimuli such as snapshots of hands detached from background). However, a few recent studies accounting for the role of contextual cues, motivational states, and social factors, have challenged this view by showing that motor resonance is not completely impervious to top-down modulations. A debate is still present. We reasoned that motor resonance reflects the inner replica of the observed movement only when its modulation is assessed during the observation of movements in isolation. Conversely, the presence of top-down modulations of motor resonance emerges when other high-level factors (i.e., contextual cues, past experience, social, and motivational states) are taken into account. Here, we attempt to lay out current TMS studies assessing this issue and discuss the results in terms of their potential to favor the inner replica or the top-down modulation hypothesis. In doing so, we seek to shed light on this actual debate and suggest specific avenues for future research, highlighting the need for a more ecological approach when studying motor resonance phenomenon.

## Introduction

**Figure 1:**
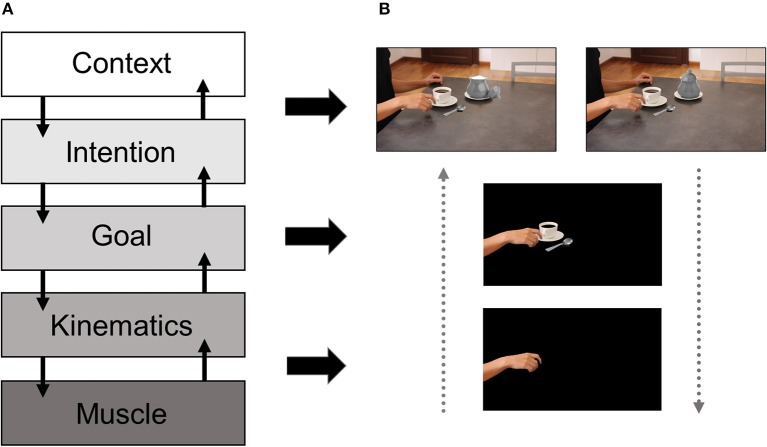
Hierarchical model of action representation and examples of stimuli varying in their level of complexity. The left panel **(A)** shows the different levels of description at which actions can be understood. Initially, this motor hierarchy included the muscle, the kinematics, the goal and the intention levels (Hamilton and Grafton, 2007). More recently, context has been proposed as a higher top-down level assisting intention coding under situations of perceptual ambiguity (Kilner et al., 2007). The right panel **(B)** shows different versions of the same stimulus varying in the amount of information provided to the observer, from kinematics only to the presence of contextual cues. In the depicted example, without the broader context including the sugar pot either opened or closed, it would be difficult to disambiguate if the precision grip used by the model is directed toward the spoon, hence cueing to the intention of pouring sugar in the coffee or, alternatively, to the handle of the cup, thus cueing to the intention of drinking coffee.

Understanding others' intentions via observing their actions is critical for social cognition. This ability is considered to be supported by the so-called mirror neuron system (MNS, Rizzolatti et al., 1996), a collection of fronto-parietal regions which become active during both observation and execution of similar actions. While initially discovered in monkeys (di Pellegrino et al., 1992), MNS-like activity has also been shown in humans by means of higher activation in the primary motor cortex (M1) in response to observed human actions, as compared to action unrelated control conditions (Fadiga et al., 2005). Here, we refer to motor resonance as an index of mirror-like activity, which reflects an enhancement of M1 corticospinal excitability (CSE) during action observation (AO). A classical way to measure motor resonance relies on the use of motor-evoked potentials (MEPs) induced with transcranial magnetic stimulation (TMS) in peripheral muscles, which reflect the level of CSE resulting from M1 stimulation. Despite the agreement in considering motor resonance as a marker of MNS-like activity, its specificity and meaning is still unclear (D'Ausilio et al., 2015). Indeed, there is an ongoing debate as to whether motor resonance would reflect an automatic replica of the observed movement, thus mirroring the kinematic features of the observed action, or rather its final goal and overarching intention, thus reflecting a more flexible process. Briefly, when considering action coding, different hierarchical levels can be identified (see Figure 1A): (i) the muscle level, which codes for the pattern of muscular activity required to execute the action; (ii) the kinematics level, which maps the movements of the effectors in space and time; (iii) the goal level, which includes the short-term transitive or intransitive aim; and (iv) the intention level, which includes the long-term purpose behind the action (Hamilton and Grafton, 2007). Interestingly, it has been proposed that, in addition to these well-known four levels, context can be seen as a fifth top-down level guiding action comprehension under situations of perceptual uncertainty (Kilner et al., 2007, Kilner, 2011). In this view, contextual cues and prior knowledge would aid action recognition by signaling which intentions are more likely to drive upcoming actions given the information present in the environment, forming the basis to estimate lower-level aspects of action representation (i.e., kinematics).

Here, we propose that motor resonance mirrors low-level aspects when stimulus complexity is low or when no additional manipulations on the observer's expectations or motivational states are present in the task at hand. Alternatively, but not exclusively, high-level factors can intervene and modulate motor resonance when more complex stimuli and/or task manipulations are taken into account. By stimulus complexity, we refer to the amount of information available to the observer in different AO paradigms (see Figure 1B). Basically, stimulus complexity can span from a low complexity degree conceivable when hand/arm movement kinematics is presented in isolation to a higher complexity degree when movement kinematics can be dissociated from its goal or underlying intention. Lastly, the highest complexity degree can be conceived when stimuli are presented in wider social scenarios including objects, agents, and their possible interactions. Thus, in the present paper the term “complexity” is strictly related to the ecological dimension of the stimulus.

Claiming that low-level or rather high-level aspects are reflected in the observers' motor system would imply the possibility of finding differential patterns of responses in CSE. Generally, motor resonance can be featured by, at least, three core elements: muscle-specificity, direction, and timing of the modulation (see Naish et al., 2014 for a review). First, *muscle-specificity* during AO implies a change in the activation of the cortical representation of the muscles that are specifically involved either in the observed action or in its execution (Fadiga et al., 1995; Strafella and Paus, 2000). In keeping with the lateralization patterns for motor control, the observed body part is mapped onto the contralateral M1 region controlling it (Aziz-Zadeh et al., 2002). Second, the *direction of the modulation* could consist in an increase or a decrease in CSE during AO, which could mirror, or not, the modulation of muscle activation during action execution. Importantly, even if in most of the cases AO mirroring is reflected into an increase in CSE with respect to a baseline level, CSE decreases during AO have also been reported (Gangitano et al., 2004; Janssen et al., 2015; Amoruso and Urgesi, 2016). Whatever the direction of this CSE modulation is, these effects might result from the simultaneous contribution of excitatory and inhibitory mechanisms, involving premotor and primary cortices (Vigneswaran et al., 2013, Kraskov et al., 2014; Gueugneau et al., 2016). Differences in TMS protocols and experimental designs may unveil excitatory or inhibitory mechanisms, possibly reflecting covert imitation or the withholding of unintended overt movement during AO. Nevertheless, it is worth mentioning that the direction of CSE modulation reported in each study may have a different meaning, mainly depending on the selection of the baseline condition with which the effects are compared and the way data are normalized (Naish et al., 2014).

Lastly, the *timing of the modulation* depends upon the delay at which motor resonance occurs, with respect to action observation onset. In this vein, a CSE modulation occurring immediately after the perception of an action is taken as a marker of the automatic simulation and the faithful covert replica of the observed movement. Time-locked modulations can be assessed by recording MEPs at different time points during AO (Sartori et al., 2013; Cavallo et al., 2014; Mc Cabe et al., 2015; Amoruso et al., 2016). Based on the presence of somatotopic, direction-specific, or time-locked effects, motor resonance has been conceived as an inner replica of the observed movements (Naish et al., 2014), thus reflecting the automatic mapping of low-level motor aspects. Conversely, when movements are observed embedded in a richer context (i.e., where the information at hand allows representing the action at a higher-level such as the goal one), the observed kinematic seems to be mirrored in a less specific fashion. Thus, motor resonance becomes prone to top-down modulations and switches from low to high representational levels. With this mini review, we sought to understand whether stimulus complexity, in combination with the timing at which indices of motor resonance are recorded, could explain why, in some cases, motor resonance corresponds to the automatic muscle-specific, direction-dependent, and time-locked simulation of the observed movement, while in other cases high-level factors and top-down modulations make motor resonance a more flexible phenomenon. Given this aim, here we focus on neurophysiological studies using single-pulse TMS and measuring MEPs in combination with action observation paradigms. Starting from the seminal studies finding a close correspondence between CSE modulation and low-level features during AO, we moved to examine those studies addressing how this low-level mirroring could be affected by, or dissociated from, high-level features (e.g., the goal of an action), and finally to more recent studies exploring the occurrence of top-down modulation during AO. Details of the revised literature are reported in Supplementary Table 1.

## From Kinematics to Goals: Mirroring Actions at Different Levels of Representation

After Fadiga et al. (1995)'s seminal study showing motor resonance during AO in humans, Gangitano et al. (2001) reported a muscle-specific increase in CSE for finger opening movements which reflected the increased amount of aperture coherent with the observed movement phase. Phase-and muscle-specific modulations were further shown during the observation of either transitive (Montagna et al., 2005) or intransitive movements (Borroni et al., 2005) and during the observation of possible and impossible finger movements (Romani et al., 2005). Moreover, enhanced muscle-specific motor resonance for abduction finger movements is influenced by onlooker's hand orientation (Maeda et al., 2002) and position, congruently with the maximal activation of the same muscles during action execution (AE) under different postures (Urgesi et al., 2006). Importantly, while claiming for a muscle specific modulation, only few of these studies assessed the correspondence between the observed modulation and the actual involvement of the same muscle during action execution (Fadiga et al., 1995; Borroni et al., 2005; Montagna et al., 2005; Romani et al., 2005; Urgesi et al., 2006).

Nevertheless, the finding of CSE modulations for intransitive actions challenged the initial view, grounded on monkey studies, that the MNS primarily encodes the action goal rather than the observed movements (Gallese et al., 1996; Umiltà et al., 2001). While favoring the low-level hypothesis, according to which motor resonance is a covert mimicry of the observed movement, these findings opened the debate about which action level is motor resonance coding for. However, these previous studies did not allow dissociating the contribution of kinematics vs. goal encoding, leaving the controversy about the level of action representation unsolved. To clarify this issue, Cattaneo et al. (2009) designed a paradigm to dissociate the two aspects. Participants observed a hand manipulating normal or reverse pliers without an evident goal or, alternatively, with the goal to grasp an object. Observing closing vs. opening hand movements when no goal was present (i.e., closing the normal or opening the reverse pliers without grasping an object) resulted in stronger activation of the opponens pollicis (i.e., a muscle involved in thumb opposition for finger closing movement), thus mirroring the hand movements. Conversely, when a goal was present, motor resonance no longer reflected the observed hand movements but rather the motor goal (achieved by opposite movements of the hand manipulating the pliers). While suggesting an incorporation of the tool into the body representation and the possibility for motor resonance to be shaped accordingly, these results supported the influence of high-level features on motor resonance.

Although divergent findings have also been found (Cavallo et al., 2012), the integrated contribution of kinematics and goal coding has been widely supported. For instance, Mc Cabe et al. (2015) found that observing the grasping of small or big objects induced both a kinematic- and a goal-specific modulation of CSE, but only when the goal could be inferred from the initial part of the movement. Conversely, when the motor goal was ambiguous (i.e., switched online between objects) and the goal could no longer be inferred, CSE modulation mirrored low-level kinematics only. In this study, the amount of visual information provided and the time at which motor facilitation was recorded were crucial in biasing the modulation toward either the low- or the high-level CSE modulation view. Thus, when the complexity of the visual stimulus increases (i.e., the information provided allows representing the action at a higher-level such as the goal one), motor resonance switches from the low to the high representational level. In the absence of this information, motor facilitation mimics the observed kinematics.

In line with this view, Betti et al. (2015) found that the observation of index finger movements, in isolation, triggered muscle-specific CSE modulations. However, when similar kinematics were then used in a symbolic way to simulate an action typically performed with the leg (i.e., a soccer penalty kick), CSE modulations were observed in both hand and leg muscles, suggesting a simultaneous representation of action meaning and movement kinematics in the observer's motor system. Differently stated, as complexity in the observed stimulus increased, motor resonance switched from low- to high-level mapping, showing a generalization between muscles. Likewise, Finisguerra et al. (2015) found that, during the observation of intransitive closing hand movements, an increase in motor resonance for a forearm flexor muscle (involved in hand closing) generalized across other effectors involved in performing closing movements (i.e., eyelid, mouth). Even if no modulation in stimulus complexity was present, it is likely that the common action goal, which could be inferred from the observed set of stimuli, contributed to this high-level mapping of action meaning. Additional evidence comes from Senna et al's study (2014), in which familiarity with an observed action elicited a shift from lower- to higher-level motor mapping. Specifically, participants viewed typical hand actions (i.e., grasping a pencil) and typical foot actions (i.e., pressing a pedal) that could be performed by either a hand or a foot effector, resulting in familiar or unfamiliar actions if performed with the typical or an atypical effector, respectively. Observing unfamiliar actions resulted in an effector specific modulation of hand and foot CSE. Conversely, during the observation of familiar actions, CSE modulation of the muscle involved in the represented action generalized across both effectors. This evidence hence suggests that actions can be coded either in a somatotopic low-level or in a goal high-level fashion, depending on the familiarity with the observed action.

## From Muscle and Force Requirements to Intention Representation

Beyond these well-detectable kinematic features mirroring the phase, the type, the extent and the effector of an observed action, other studies sought to understand whether motor resonance could be sensitive to less salient changes in kinematic signals, such as muscle involvement and force requirements during AE. In this vein, Alaerts et al. (2009) aimed to disentangle the contribution of muscle and movement direction coding to motor resonance. Participants were asked to observe a model performing upward movements of the wrist. These movements could require the involvement of the flexor or the extensor muscle, depending on the starting position of the model's hand (palm-up or palm-down). Importantly, participants could keep their hand in a palm up or palm down position so that their posture could be congruent or incongruent with respect to the posture of the model. Muscle-specific mapping would imply that the increased activation of the model's muscle during the observation of upward wrist movement led to a muscle-specific CSE modulation in the observer, independently from the observer's posture. Conversely, direction-specific mapping would imply an activity modulation specific for the muscle that allows for the execution of an upward movement depending on the posture of the observer, thus independently from the muscle activated in the model's movement. Given that they only found an interaction between the observer's muscle and the model's movement, CSE facilitation was thought to be independent from the muscle involved in the observed movement. Importantly, even if no significant effects or interactions with observer's posture were found, the muscle-specific facilitation was maximal during congruent postures (i.e., when the muscular and the directional parameters overlapped), and became less evident, albeit still present for one muscle, when the muscular and directional features were discordant.

Interestingly, in a subsequent study, Alaerts et al. (2010a) found that motor resonance was congruent with the degree of muscular involvement in AE. Indeed, while keeping the observed action (i.e., grasping-and-lifting-the-object) constant but changing the object weight, greater facilitation for heavy object lifting than light object lifting was found. Moreover, the observation of either precision or power grasp-to-lift resulted in a weight-dependent muscle-specific motor resonance. Despite this modulation potentially resembling the gradually increasing activation of these muscles in AE, these findings only partially support the low-level coding hypothesis. Indeed, in these studies, information about object weight could be easily inferred from object appearance. Thus, as the authors themselves suggested, their stimuli did not allow clarifying whether the force-related effects on motor resonance were driven by low-level kinematics observation or by high-level expectations triggered by object properties. In a follow-up study (Alaerts et al., 2010b), the role of different visual cues (i.e., kinematic profile, hand contraction, and intrinsic object properties) contributing to motor resonance during object lifting were separately tested. Even if the weight information carried by movement kinematics and hand contraction modulated motor resonance in the opponens pollicis and extensor carpi radialis muscles, a conflict between movement kinematics and object appearance reduced the weight-dependent modulation in the opponens pollicis. While supporting a low-level coding of observed action, we interpret these findings as evidence for a contribution of observers' expectations (e.g., triggered by object properties) in interfering with the weight-dependent motor resonance modulation. Indeed, findings from a following study support this view. For instance, in Senot et al. (2011), the observation of lift-to-place actions directed to heavy or light objects led to a weight-dependent activation of the FDI muscle, regardless of object intrinsic properties (i.e., when the content of a bottle was visible or hidden from view), suggesting that movement kinematics was enough to modulate CSE. However, when explicit semantic cues, either congruent or incongruent with respect to the actual object weight, were provided by verbal labels, the weight-dependent modulation ceased. Unfortunately, the possibility that the limited sample size of participants (Alaerts et al., 2009; Senot et al., 2011) may have led to underpowered studies unable to unmask the interactive effects, cannot be excluded and caution is needed when drawing conclusions from this data.

The relevance of low- and high-order factors in shaping motor resonance was further confirmed by a set of subsequent studies dealing with object weight discrimination (Tidoni et al., 2013; Finisguerra et al., 2018). In Tidoni et al. (2013), motor resonance modulations were assessed during the observation of reach-to-lift actions of either light or heavy objects that could be performed with a genuine or a deceptive intention. Even if motor resonance was greater for heavy than light object grasping, the authors also found that observing deceptive actions facilitated FDI CSE more than observing genuine actions, regardless of object weight. However, this study did not allow for ascertaining whether low-level (i.e., the altered kinematic patterns required to deceive the observer) or high-level (i.e., the deceptive intention) features explained the observed effect. Thus, in a subsequent study, Finisguerra et al. (2018) sought to solve this question. By independently manipulating the actor's deceptive or genuine intentions and kinematic alterations, the authors found that while the observation of deceptive actions facilitated CSE in a muscle-independent fashion, the observation of kinematic alterations driven by genuine intentions induced a muscle-specific CSE inhibition, which resembled the pattern of muscle activation during AE in the same condition. Overall, both low-level and high-level features were mirrored into the observer's motor system in a dissociable fashion.

## Top-Down Contextual Modulations During Action Observation

Mounting evidence suggests that motor resonance can be modulated by a wide range of high-level contextual factors. Indeed, human actions do not occur in isolation but rather embedded in internal and external contexts.

On the one hand, when considering studies reporting top-down modulations associated to internal factors, such as individual personality traits and temperament, it appears that they play a critical role during AO. For instance individuals with an increased level of harm avoidance personality trait, which is mainly characterized by excessive worrying, exhibited reduced motor resonance during the observation of immoral as compared to neutral actions (i.e., stealing a wallet vs. picking up a notepaper, respectively) containing similar kinematics (Liuzza et al., 2015). Likewise, Craighero and Mele (2018) reported that the observation of an agent performing an action with negative (i.e., unpleasant) consequences on a third person results in decreased motor resonance as compared to the observation of actions underpinning positive and neutral intentions with equal kinematics. Nevertheless, no correlations were observed in this latter case between the levels of harm avoidance personality trait and the CSE effect.

The observer's current state also plays a critical role during AO. For instance, Hogeveen and Obhi (2012) found that, during the observation of human and robotic actions (i.e., a human hand or grabber reaching tool squeezing a ball, respectively), participants previously involved in a naturalistic social interaction with the experimenter, showed increased CSE for the observation of human actions as compared to robotic ones. This effect was absent in those individuals not previously engaged in the social interaction, with human and robotic actions triggering similar levels of CSE. In a more recent study, Hogeveen et al. (2014) found that CSE while observing a hand squeezing a ball becomes differentially modulated after participants being exposed to a low- or a high-power induction priming procedure (i.e., recalling a memory in which someone else had power over observers or in which the observer had power over someone else). Participants in the high-power group showed less motor resonance facilitation relative to the low-power group, suggesting that people in positions of power display reduced interpersonal sensitivity and diminished processing of social input. In sum, these studies suggest that prior naturalistic social interactions of different kinds modulate motor resonance for subsequent action observation.

Another factor that has been reported to modulate AO is prior experience and/or familiarity with the observed action (Aglioti et al., 2008; Candidi et al., 2014). Candidi et al. (2014) showed to expert pianists and naïve controls videos displaying a professional pianist that could perform fingering errors while playing musical scales. Even though non-pianist controls were visually trained to recognize the errors in the videos, only piano experts showed a somatotopic modulation in the abductor pollicis brevis muscle (i.e., the muscle involved in the execution of the piano fingering errors), with increased MEP amplitudes 300 ms after error onset. Overall, this suggests that prior motor (but non-visual) experience is necessary for motor resonance. A similar increase in MEP amplitudes in the abductor digiti minimi muscle have been reported in basketball experts while observing “out” as compared to “in” shots in a basket (Aglioti et al., 2008). While demonstrating that prior motor experience provides a fine-grained simulative error monitoring system to evaluate others' movements, these studies suggest that high-level information (i.e., movement correctness) can influence motor resonance during AO.

On the other hand, parallel top-down modulations have been observed when considering external contextual factors. In a series of studies, Amoruso et al. (Amoruso and Urgesi, 2016; Amoruso et al., 2016) explored the role of contextual information in modulating action coding at lower levels of representation (i.e., muscle and kinematics). CSE was measured while participants were asked to observe actors performing everyday actions embedded in congruent, incongruent or ambiguous contexts, and to recognize actor's intention. Context-action congruency was manipulated in terms of compatibility between grasping kinematics and action setting. For instance, within a breakfast scenario (e.g., a cup full of coffee), the actor could grasp the cup by its handle with a precision grip (congruent condition) or with a whole-hand grip from the top (incongruent condition). Ambiguous contexts (i.e., a cup half full of coffee) where different types of actions were equally plausible were also used. As compared to the neutral condition, the congruence between the movements and the context increased CSE at early stages (~240 ms after action onset), while incongruence between them resulted into a later inhibition (~400 ms) for the FDI muscle, which is involved in reaching-to-grasping movements. Crucially, the different time course and direction (i.e., facilitation vs. inhibition) of the observed effects suggests that they stem from partially independent mechanisms, with the early facilitation directly involving simulative motor resonance through the classical AO network, and the later inhibition recruiting structures outside of this network conveying information about the intention estimated from the context.

Additional evidence from a role of top-down contextual modulation on motor resonance comes from two recent studies. In the first one, Riach et al. (2018) used a similar logic but introduced a baseline condition in which actions were observed without a context. Similar to Amoruso et al. (2016) findings, observation of actions within congruent contexts (i.e., pinching a sponge in a kitchen background) facilitated FDI CSE as compared to baseline. No modulation of CSE resulted from AO in incongruent contexts. In a second study by Cretu et al. (2019), participants observed either full or occluded videos of an actor grasping and lifting a jar using a precision or a whole-hand grip. Color cues preceded observation trials and were manipulated in terms of their informativeness in predicting the upcoming action. Overall, the authors found that even in the absence of movement kinematics (i.e., occluded condition), contextual reliable cues were sufficient to trigger a muscle-specific response in the observer. Nevertheless, when presenting both sources of information together (i.e., kinematics and context), CSE facilitation became stronger than when either source was presented alone. These findings support the view that motor resonance triggered by observed kinematics and top-down contextual information interact in the observer's motor system.

Regarding the inhibitory effects on CSE reported for contextual conflicting information (Amoruso and Urgesi, 2016; Amoruso et al., 2016), similar findings were reported by Janssen et al. (2015). They showed that incongruence between an action specified by a prior symbolic cue (i.e., an arrow indicating the requirement of a whole-hand grip) and the observed action (i.e., movement implying a precision grip) leads to a reduction in motor resonance for the observed action, with CSE replicating the motor pattern of the action specified by the prior cue. Likewise, Mattiassi et al. (2014) found that the observation of hand movements preceded by an incongruent masked prime (e.g., a different hand movement) decreases motor resonance responses in a comparable fashion.

Finally, another aspect that has been shown to modulate motor resonance is the social nature of the context. Sartori et al. (2013) recorded MEPs at different time points while participants observed action sequences that could call for a complementary response (or not) depending on the context. Specifically, participants were asked to observe videos of a model grasping a spoon or a thermos to pour sugar or coffee into three cups/mugs located on a table next to her, using a precision or a whole-hand grip, respectively. After this, the model poured sugar/coffee into a fourth cup/mug that was located far away from her and, thus, closer to the participant observing the video. Crucially, a person wanting to pick-up that fourth cup/mug would need to use either a precision or a whole-hand grip to do it. Interestingly, the authors found that, while at the beginning of the video, when the context called for an imitative action, participant's CSE reflected symmetrical motor resonance for actions performed with the thermos in the abductor digiti minimi (a muscle mostly involved in whole-hand grasping), during the last part, it shifted to simulate the complementary response, with decreased abductor digiti minimi CSE for actions evoking a complementary response using a precision grip (i.e., grasping the cup). The reverse pattern was found for the spoon-related action. These findings point to the fact that motor resonance can be modulated (i.e., shift from emulation to reciprocity) depending on the social context in which it takes place, in agreement with a flexible view of this phenomenon (Heyes, 2010, Keysers and Gazzola, 2014).

Overall, a final consideration integrating both internal and external factors should be made. It has been shown that when contextual cues are not available, information from observed movement kinematics forms the basis for action comprehension (Soriano et al., 2018). However, it is also true that not all individuals are able to detect subtle kinematic differences from observed movements (Naish et al., 2013) and this ability may vary from one individual to another. Thus, assessing individual personality traits such as observer's visual processing style during the experiments may be a useful approach to better understand the interaction between internal and external factors during motor resonance.

## Conclusions and Future Directions

All in all, despite task and stimulus-related differences across the reviewed studies, a clear picture emerges, suggesting that motor resonance is not an automatic response reflecting the inner replica of the observed movement but rather a dynamic and flexible phenomenon, prone to modulations of internal and external factors.

First, as soon as the experimental design allows for a dissociation between the kinematics and the goal profiles or when the complexity of the stimuli increases, a transition from low- to high-level mapping becomes evident (Cattaneo et al., 2009; Mc Cabe et al., 2015). Similarly, when considering studies on object weight discrimination, motor resonance mirrors force-related modulations only when no additional information or experimental manipulations about object intrinsic and extrinsic properties are present. When a conflict between the observed action and the object-based expectations takes place, or when these expectations are diverted through deceptive intentions, high-level rather than low-level features shape motor resonance (Tidoni et al., 2013; Finisguerra et al., 2018).

Second, studies reporting motor resonance modulations triggered by contextual cues during action recognition suggest the involvement of at least two distinct mechanisms of motor resonance regulation. Specifically, when perceptual movement kinematics and external contextual information are compatible and point to the same underlying motor intention, a muscle-specific facilitation of CSE becomes evident (Amoruso et al., 2016; Riach et al., 2018; Cretu et al., 2019). Conversely, when external contextual-cues are not compatible with the observed kinematics, motor resonance becomes suppressed/reduced (Janssen et al., 2015; Amoruso and Urgesi, 2016). Interestingly, an analog suppression is observed when the action due to its immoral or negative valence conflicts with the observer's personality (Liuzza et al., 2015; Craighero and Mele, 2018).

Third, regarding the notion of stimulus complexity previously described in the introduction, it emerges that studies reporting low-level motor coding have mostly considered movement in isolation (i.e., using snapshots of hands detached from background). However, when movements are observed in more ecological settings, motor resonance integrates high-level aspects of action representation on its mirroring pattern.

When considering timing, only few of the reviewed studies explored the modulation of CSE during AO at different time-points depending on the phase of the movement (Cavallo et al., 2012; Sartori et al., 2013; Candidi et al., 2014; Mattiassi et al., 2014; Betti et al., 2015; Janssen et al., 2015; Mc Cabe et al., 2015; Amoruso et al., 2016; Cretu et al., 2019). Overall, those studies tracking the time-course of top-down modulations indicate that they arise from around ~240–300 ms post movement onset but not before. This is in line with a recent two-stage model (Naish et al., 2014; Naish and Obhi, 2015) suggesting that muscle-specific and high-level modulations on M1 responses occur in a later time-window, from ~200 ms after movement onset onwards.

Nevertheless, other potential explanations can account for the alternative mapping of low- and high-level features during AO. For instance, starting from motor control model of action, D'Ausilio et al. (2015) have proposed that this fragmentation, in the representation of low- and high-level aspects during AO, mirrors the synergistic organization of the motor system. As such, the functional output of the motor system can be better extrapolated from TMS-induced Motor Evoked Kinematics MEKs than by single-muscle MEPs (Bartoli et al., 2014; Finisguerra et al., 2015; Fricke et al., 2017; Hilt et al., 2017). Differently from MEPs, MEKs are thought to measure the effect of the synergistic activity of multiple muscles underlying the execution of coordinated movements, under the influence of intracortical, corticospinal, spinal, and peripheral factors (Fetz et al., 2002; Hilt et al., 2017). Thus, methodological aspects could also explain differences in the revised literature and future research addressing this issue is clearly required.

In view of this, some considerations should be made about potential methodological limitations identified in the reviewed studies and what could be improved. A first aspect that emerges is that only few of these studies recorded EMG also during AE and not solely during AO. Indeed, the absence of AE evidence narrows the possibility of claiming for a muscle-specific effect. Second, a proper control condition is not always used in these studies, which makes it difficult to ensure that observed effects truly correspond to the experimental manipulation or to other confounding factors. Despite a few cases lacking a baseline measure (Montagna et al., 2005; Cattaneo et al., 2009; Alaerts et al., 2010b; Senot et al., 2011), most of the revised studies report AO effects on CSE with respect to a baseline level. However, baseline levels were acquired under diverse conditions, with MEP recorded during eye-closed conditions or during observation of a black screen, a fixation cross, geometrical shapes, moving stimuli, static object, and static hand. Nevertheless, from all these heterogeneous measures, only the static hand condition takes into account the activation that the observation of a biological effector can induce *per se* on CSE. At the same time, when the observation of a static hand is used as a baseline measure, the possible effects induced by the implied motion present in the stimuli (Urgesi et al., 2010) can lead to a suppression rather than to an enhancement in CSE during the AO conditions vs. the baseline level (Mc Cabe et al., 2015). Importantly, the selection has to be driven by the experimental stimuli used in the main protocol and the experimental question. Following the rationale underscored by this mini review, a relaxed hand detached from the context would be the appropriate control condition to assess the transition from the lowest to the highest level in action representation depending on stimulus complexity.

Finally, we consider that the use of designs allowing dissociating motor coding at different levels of representation as well as controlled experimental conditions varying the amount of stimulus complexity would also be of great help to shed light on this issue. More specifically, this proposal opens future avenues to empirically test a set of hypotheses on how motor resonance responses may vary (or not) at different levels of action representation (i.e., muscle, kinematics, goals/intentions) when manipulating external and internal factors. For instance, an ideal experimental approach would comprise a battery of tasks varying the degree of the information provided to the observers (e.g., from frames showing hand movement kinematics in isolation to frames showing them in relation to objects and, finally, embedded in a wider social scenario). This would help to disentangle how external factors modulate motor resonance within the same set of participants and tasks in a controlled fashion. Furthermore, by measuring participants' individual traits, correlations between their personality profiles and their motor and behavioral responses can be performed in order to explore the possible influence of internal factors. For instance, Amoruso et al. (2018) have recently found that individuals with a high amount of autistic traits (i.e., social deficits and greater detail-processing style) are more impaired in suppressing motor resonance when a mismatch between kinematics and context occurs, pointing to difficulties in their integration. Shedding light on this latter aspect would allow for a better understanding of its functional role, not only in neurotypical individuals but also in psychiatric disorders such as autism, in which abilities grounded in motor resonance are critically impaired.

## Author Contributions

LA and AF conceived the study, performed the research, wrote, and revised the manuscript. All authors approved the final version of the manuscript.

### Conflict of Interest

The authors declare that the research was conducted in the absence of any commercial or financial relationships that could be construed as a potential conflict of interest.

## Abbreviations

AE: Action Execution
AO: Action Observation
CSE: Cortico Spinal Excitability
FDI: First Dorsal Interosseous
M1: Primary motor cortex
MEP: Motor Evoked Potentials
MNS: Mirror Neuron System
TMS: Transcranial Magnetic Stimulation.
