# Supplementary material for: Low or High-Level Motor Coding? The Role of Stimulus Complexity
Source: Front Hum Neurosci. 2019 Oct 11;13:332. doi: 10.3389/fnhum.2019.00332 (PMC6798151; doi:10.3389/fnhum.2019.00332)
Supplement: Supplementary file 1 [file Table_1.pdf]

STUDY	METHOD					RESULTS	
	Sample N (M), age	Observed action/ Experimental manipulation	Baseline condition /data normalization	Muscle (s)	Type and time of stimulation	CSE Effects during AO	Effects
**Fadiga et al., 1995**	12 pp	Gasping movements<br>Arm movements<br><br>+ EMG recording during AE of grasping and arm movements.	Static object<br>Dimming of light stimulus<br><br>Z-scores	EDC<br>FDS<br>FDI<br>OP	Sp TMS (100% MT)<br>Just before the end of stimulus presentation or between light presentation and dimming in the fourth conditions.	EDC, FDS, FDI CSE: ↑ during the observation of grasping action and arm movements vs static object observation and dimming light detection;<br>OP CSE: ↑ during the observation of grasping action vs static object observation, dimming light detection and arm movement	Muscle specific CSE facilitation during movement observation, following EMG activation during AE
**Gangitano et al., 2001**	8 pp<br>(29.7 ± 4.3 yr)	Reaching and grasping a ball with a PG (five levels of finger aperture: 0°, 28°, 53°, 61°, 30°).	Blank screen	FDI<br>APB	SpTMS (SI NA)<br>For each level of finger aperture (500, 1000, 2000, 3000, 3500ms) and during blank screen (5500 ms) from video onset.	FDI CSE:<br>-↑ during PG vs blank;<br>-↑ peak at 3000ms (max aperture);<br>-phase dependent modulation according to Index finger (vs Thumb) displacement.<br><br>APB CSE: ↑ during PG vs blank	Phase and muscle dependent mirroring of index finger aperture during PG AO
**Montagna et al., 2005**	5 pp<br>(1 male, 22-44 yr)	Reaching and grasping a ball with a WHG (four levels of finger aperture: mid hand opening, end of hand opening, mid hand closing on the ball, fingers contact with the ball) + EMG recording during AE.	None	FDS<br>FCR<br>FDI	Sp TMS (110% MT)<br>For each level of finger aperture (2500, 3000 ms, 3500 ms, 4300 ms) from video onset.	FDS CSE: ↑ during opening phase<br>FDI CSE: ↑ during closing phase	Opposite CSE modulations of the two muscles, that followed the EMG activation in AE
**Borroni et al., 2005**	5 pp from a bigger sample of 12 pp<br>(7 male, 20-45yr)	Flexion-extension wrist movements cycles (1Hz) + EMG recording during AE.	Flexion and extension (1.6 Hz) wrist movements;<br>Oscillating (1 Hz) platform<br><br>Normalization to participant's average	FCR<br>ECR	SpTMS (110% MT)<br>At 0, 200,400, 600, 800 ms corresponding to 5 different angular positions during live AO.	FCR and ECR CSE fitted the sine wave function with the same period of the observed movement	Phase and muscle dependent mirroring for intransitive movement, following EMG activation in AE

STUDY	METHOD					RESULTS	
	**Sample N (M), age**	**Observed action/ Experimental manipulation**	**Baseline condition /data normalization**	**Muscle (s)**	**Type and time of stimulation**	**CSE Effects during AO**	**Effects**
**Romani et al., 2005**	EXP1: 11 pp (3 male, 30.0 ± 4.9 yr)	Bio-mechanically possible or impossible: -index finger abduction/adduction movements;	Eyes closed Static hand	FDI ADM	SpTMS (130% MT) At randomized intervals between 1500 ms and 2500 ms from video onset.	FDI CSE: ↑ during either possible and impossible movements (vs no observation or still hand observation baselines); ADM CSE: no AO effects;	Muscle specific CSE facilitation for the muscle maximally involved in the execution of the OA, following EMG activation during AE, for either possible and impossible movements
	EXP2: 14 pp (6 male, 23.6 ± 3.1 yr)	-little finger abduction/adduction movements;	Contracting/ expanding (1Hz) circle	FDI ADM		ADM CSE: ↑ during either possible and impossible movements (vs expanding-contracting circle baseline); FDI CSE: no AO effects;	
	EXP3: 11 pp (3 male, 26.4 ± 4.7 yr)	-index finger extension/flexion movements;	Static hand	FDI ADM		FDI and ADM CSE: no AO effects;	
	EXP4: 15 pp (4 male, 27.1 ± 6.1 yr)	-index finger extension/flexion movements.	Static hand	FDI EIP		EIP CSE: ↑ during either possible and impossible movements (vs still hand observation baseline); FDI CSE: no AO effects.	
	EXP5: 6 pp (2 male, 24.6 ± 10.5 yr)	+ EMG recording during possible index or little finger abduction/adduction movements or index finger extension/flexion movements.					
**Maeda et al., 2002**	10 pp (4 male, 29.8 yr)	While keeping hand in a position that matched the observed *away* hand orientation<br>- away thumb abduction;<br>- away index finger abduction;<br>- away index finger vertical movement;<br>- toward thumb abduction;<br>- toward index finger abduction;<br>- toward index finger extension/flexion movement.	Eyes closed<br><br>Percentage change from baseline	APB FDI	SpTMS (120% MT) 4 to 6 s from video onset.	APB CSE:<br>- ↑ during thumb vs index abduction condition;<br>-↑ during away (compatible) vs toward orientation;<br><br>FDI CSE: no effects of thumb orientation;<br><br>FDI CSE: ↑ during index finger vs thumb abduction condition for away (compatible) vs toward orientation;<br>APB CSE: no effects of index finger orientation;<br><br>FDI CSE: ↑ during index finger extension/flexion condition for away (compatible) vs toward orientation.	Facilitatory muscle specific and orientation sensitive effects for the muscle involved in the OA.

STUDY	Sample N (M), age	Observed action/ Experimental manipulation	Baseline condition /data normalization	Muscle (s)	Type and time of stimulation	CSE Effects during AO	Effects
	METHOD					RESULTS	
**Urgesi et al., 2006**	EXP 1: 16 pp (9 male, 21.9 yr)<br><br>EXP 2: 12 pp (4 male, 23.5 yr)<br><br>EXP 3: 8 pp (2 male, 23.9 yr)	Palm up and palm down:<br><br>-index finger and little finger abduction/adduction movements (hand kept palm down);<br><br>-index finger and little finger abduction/adduction movements (hand kept palm up);<br><br>+ EMG recording during AE.	Eyes closed<br>Static hand<br><br>Z-scores	FDI<br>ADM<br><br>+ ECR	SpTMS (130% MT)<br>At 660 and 990 ms before the end of 3300 ms videos.	FDI CSE:<br>-↑ during index finger abduction abduction/adduction movements (vs still hand baseline or vs little finger movements),<br>-nonsignificant effect of posture nor interaction<br>ADM CSE: nonsignificant effects of movement, posture nor interaction.<br><br>FDI CSE: nonsignificant effects of movement, posture nor interaction.<br>ADM CSE: ↑ during little finger abduction/adduction movements (vs still hand baseline or vs index finger movements), nonsignificant effect of posture nor interaction.	Facilitatory muscle specific effects of movements in AO compatible with the maximal activation of the muscles under different postures in AE, following EMG activation during AE
**Catteneo et al., 2009**	EXP 1: 14pp (24-36 yr)<br><br>EXP 3: 8 pp (23-30 yr)	Opening and closing movements of the hand to open and close a normal (opened via thumb and index finger extension and closed via fingers flexion) plier, or a reverse plier (opened via thumb and index finger flexion and closed via fingers extension):<br><br>-without a goal;<br>-with a grasping goal.<br><br>After a motor training with pliers during the observation of hand manipulating the reverse pliers only:<br><br>-without a goal;<br>-with a grasping goal.	None	OP	SpTMS (SI ~ to obtain MEP amplitude of 1mV at rest)<br>Few milliseconds before maximal plier aperture or maximal plier closure.	OP CSE: ↑ during finger flexion phase vs extension for normal pliers both in the goal and no goal condition (Exp 1) and for reverse pliers in the no goal condition (Exp 1 and Exp 3);<br><br>↑ for reverse pliers in the goal condition during thumb and index extension phase (i.e., closing movement of the plier) vs flexion (Exp 1 and Exp 3).	Muscle specific modulation for observed hand movement when no goal was present; goal dependent modulation (mirroring the closing movement of the pliers) for grasping actions.

STUDY	METHOD					RESULTS	
	Sample N (M), age	Observed action/ Experimental manipulation	Baseline condition /data normalization	Muscle (s)	Type and time of stimulation	CSE Effects during AO	Effects
**Cavallo et al., 2012**	22 pp (7 male, 22.6 yr)	Grasping mothball actions through opening and closing movements of the hand to open and to close:<br>-a normal plier (opened via thumb and index finger extension and closed via fingers flexion);<br>-a reverse plier (opened via thumb and index finger flexion and closed via fingers extension);<br>-a magnetic pliers (allowing the grasping goal via hand and pliers opening movements).	Fixation cross<br><br>Ratio from baseline and logarithmic transformation	OP<br>FDI	SpTMS (110% MT)<br>At:<br>-pliers-object contact-0 frame,<br>-pliers-object contact-5 frame,<br>-pliers-object contact -10 frame.	No differences in CSE during AO vs baseline.<br><br>OP CSE: ↑ during closing movement of the hand, to close the normal plier vs opening movement of the hand to open the magnetic plier;<br>FDI CSE:<br>- ↑ during closing movement of the hand, to close the normal plier vs opening movement of the hand to close the reverse plier and vs opening movement of the hand to open the magnetic plier;<br>-↓ during hand opening movement with magnetic plier (no effects of movement phase for the reverse plier).	Specific modulation of CSE for hand movement rather than tool movement or distal goal
**Mc Cabe et al., 2015**	35 pp (14 male, 23.6 ± 4.5yr):<br><br>EXP1 (10 pp)<br><br>EXP2 (13 pp)<br><br>EXP3 (12 pp)	EMG recording during AO of reaching to grasp a small or a big object:<br><br>-with online action correction from large to small object;<br>-when information on action goal was provided;<br><br>-when information on action goal was not provided.	Static hand	FDI<br>ADM	SpTMS (120% MT)<br>At:<br>- 0% of movement completion,<br>-40% of movement completion (right before the online correction),<br>- 80% of movement completion (after the online correction,<br>-100% of movement completion (hand-object contact).	Increased CSE for static hand vs AO conditions;<br><br>EXP 2 (action goal information provided)<br>FDI CSE:<br>-↑ for action directed to small vs large object (consistent with AE activation), especially at hand-object time contact;<br>- ↑ only after online correction from large to small object at 80% of movement completion;<br><br>ADM CSE: no modulation;<br><br>EXP 3 (action goal information not provided)<br>FDI or ADM: no differences between to be grasped small object and online (toward small object correction) before movement completion.	CSE modulation following time-course FDI activity during AE, CSE modulation mirroring the goal only when goal information was provided,<br>CSE modulation Mirroring kinematics regardless of the available goal information
**Betti et al., 2015**	13 pp (all female, 22.5 ± 2.9 yr)	AO of:<br>-Biological index finger movement;<br><br>-Finger kick movement;<br><br>-Symbolic kick movement (simulating leg movement).	Fixation cross<br><br>Ratio from baseline	FDI<br>QF	Sp TMS (120% MT)<br>At:<br>T1, maximal flexion of the index finger,<br>T2, maximal extension of the index finger.	FDI CSE: ↑ for biological movement and finger kick AO vs QF CSE at T1;<br>QF CSE: ↑ for symbolic action vs biological movement and finger kick conditions at T1.	Muscle-specific mirroring only when the action goal cannot be represented; generalization of CSE when abstract action representation is possible

STUDY	METHOD					RESULTS	
	Sample N (M), age	Observed action/ Experimental manipulation	Baseline condition /data normalization	Muscle (s)	Type and time of stimulation	CSE Effects during AO	Effects
**Finisguerra et al., 2015**	35 pp (20 male, 24.47 ± 3.80 yr)<br><br>EXP1 (18 pp)<br><br>EXP2 (17 pp)	Opening and closing actions performed by:<br><br>-hand (homologue) and mouth (different) effectors (EXP1);<br><br>-hand (homologue) and eyelid (different) effectors (EXP2).	Nature related moving stimuli<br><br>Ratio from baseline	FDS	Sp TMS (130% MT)<br>At 90% of the whole movement duration.	FDS CSE:<br>-↑ for closing vs opening movement (EXP1, EXP2) regardless of the observed effector;<br>- ↑ for different vs homologue effector.	Direction specific motor resonance effect on MEP generalized to other effectors; mapping of complex action features (closing goal)
**Senna et al., 2014**	EXP 1:<br>10 pp (2 male, 25, 21-33, yr)<br><br>EXP2:<br>10 pp (all female, 27.7, 23-33, yr)	Action typically executed with the hand (grasping a pencil) or unspecified action (stepping over) performed with the hand or the foot;<br><br>Action typically executed with the hand (grasping a pencil) or with the foot (pressing a pedal) performed with the hand or the foot.	Static effector (hand and foot)<br><br>Ratio from baseline	FDI<br>AH	Sp TMS (120% MT)<br>At 1006 ~ 1982 ms from video onset (2.1 s total duration) immediately before the effector-object contact.	FDI CSE:<br>-↑ for action performed by the hand vs foot, regardless the type of action;<br>-↑ for grasping vs stepping over action, regardless of the effector;<br>AH CSE: ↑ for action performed by the foot vs hand, regardless of the type of action;<br><br>FDI CSE:<br>-↑ for action performed by the hand vs foot, regardless the type of action;<br>-↑ for grasping vs stepping over action, regardless of the effector;<br>AH CSE:<br>-↑ for action performed by the foot vs hand, regardless the type of action;<br>-↑ for pressing action vs grasping, regardless of the effector.	Effector specific CSE modulation for unspecified action, generalization across effectors for familiar actions
**Alaerts et al., 2009**	12 pp (5 male, 25 ± 4 yr)	Hands kept palm-up or palm down during the observation of:<br><br>-Extension and flexion movements of the wrist, in a palm down or palm up position;<br>- extension and flexion movements of the ankle, background screen.	Yellow background without limb movement<br><br>Percentage of MEP from baseline	FCR<br>ECR	2 Sp TMS (130% MT), with inter-stimulus interval > 5 sec<br>At:<br>-4 and 8 s from video onset (hand/foot reached peak UP position),<br>-3 and 9 s from video onset (hand/foot reached start DOWN position).	ECR CSE: ↑ vs FCR for upward extension movements (keeping the hand in a congruent palm down position);<br>FCR CSE: ↑ vs ECR for upward flexion movements (keeping the hand in a congruent palm up position);<br><br>ECR CSE: ↑ vs FCR for upward extension movements (keeping the hand in an incongruent palm up position);<br>FCR CSE: tendency toward ↑ vs ECR for upward flexion movements (keeping the hand in an incongruent palm down position);<br><br>ECR and FCR CSE: no modulations for foot action.	Muscle specific effects predominated over direction dependent modulation of CSE

STUDY	METHOD					RESULTS	
	Sample N (M), age	Observed action/ Experimental manipulation	Baseline condition /data normalization	Muscle (s)	Type and time of stimulation	CSE Effects during AO	Effects
**Alaerts et al., 2010a**	EXP1: 8 pp (5 male, 20-32 yr)	Reaching, PG, and lifting of light (10 g) or heavy (500 g) object;	White background	FDI	Sp TMS (120% EXP 1, 130% EXP2 MT)<br>At object lifting phase lasting:<br>~ 310 ± 70 ms for the heavy object, ~ 352 ± 90 ms for the light object (EXP 1); ~178 ms for the empty bottle, ~140 ms for the half-full bottle, ~ 180 ms for the full bottle (EXP2).	FDI CSE: ↑ for heavy vs light object (EXP1);	Weight dependent and muscle specific modulation of CSE, following EMG activation during AE.
	EXP2: 12 pp (3 male, 21-35 yr)	Reaching, WHG, and lifting of empty (0 Kg), half full (1 Kg), full (2 Kg) bottle, blank screen;	Normalization to participant's maximal MEP amplitude	OP<br>FCR<br>ECR		OP CSE: ↑ for half full or full vs empty bottle;<br>ECR CSE: ↑ for full vs empty and half full bottle;<br>FCR CSE: no modulations.	
	5 pp	+ EMG recording during AO.		OP<br>FCR<br>ECR			
**Alaerts et al., 2010b**	EXP1:<br>15 pp (6 male, 24 yr)	Kinematic profile of WHG and lifting of light (0.1 Kg) or heavy (2.1 Kg) objects, performed by a hand covered with glove and sleeve;	Normalization to participant's maximal MEP amplitude	OP<br>FCR<br>ECR	Sp TMS (130% MT)<br>At:<br>-the time interval at which vertical object displacement reached 6-8 cm (EXP1),	OP and ECR CSE: ↑for heavy vs light object;<br>FCR CSE: no modulations;	Weight dependent modulation of CSE depending on kinematic profile and isometric contraction. Importantly, expectations for intrinsic object properties partially modulates CSE
	EXP2:<br>16 pp (6 male, 25 yr)	Static image of WHG toward an object by exerting low or maximal isometric force;			-random time points during holding phase (EXP2),	OP and ECR CSE: ↑for maximal vs low isometric contraction;<br>FCR CSE: no modulations;	
	EXP3:<br>22 pp (12 male, 23 yr)	Reaching and grasping objects with different filling levels and having different weights (congruent and incongruent): high filling-heavy weight; low filling-light weight; high filling-light weight; low filling-high weight.			-random time points during lifting phase, the time interval at which vertical object displacement reached 18-20 cm (EXP3).	OP CSE: ↑for heavy vs light object, regardless of the apparent filling level;<br>ECR and FCR CSE: no modulations.	
**Senot et al., 2011**	8 pp (7 male, 19-34 yr)	Reaching, PG and lifting actions toward the cap of:<br>-transparent bottle with large or small amount of filling;<br>-opaque bottle with large or small amount of filling;<br>-opaque bottle with large amount of filling, labelled as "Heavy";<br>-opaque bottle with large amount of filling, labelled as "Light".	Z-scores	FDI	Sp TMS (120% MT)<br>At different delays after hand-cap contact:<br>in the lifting phase (78% of pulses),<br>before the lifting phase (22% of pulses).	FDI CSE:<br>- ↑ for heavy vs light object; regardless content visibility;<br>- no weight dependent modulation in the labeled conditions.	Weight dependent CSE modulation regardless the visibility of the object's content. Importantly, weight dependent CSE modulation ceased with explicit weight information

STUDY	METHOD					RESULTS	
	**Sample N (M), age**	**Observed action/ Experimental manipulation**	**Baseline condition /data normalization**	**Muscle (s)**	**Type and time of stimulation**	**CSE Effects during AO**	**Effects**
**Tidoni et al., 2013**	15 pp (9 male, 21.5 yr)<br><br>10 pp (4 male)	Reaching, grasping, lifting to place actions performed on a light (50 g) or heavy (650 g) cube by an actor providing: truthful or deceptive information about the object weight;<br>+EMG recording during AE.	Eyes closed (while imagine watching a sunset at the beach)<br><br>Logarithmic transformation and percentage change from baseline	FDI<br>FCR	Sp TMS (120% MT)<br>During the lifting and placing phase.	FDI CSE:<br>-↑ for heavy vs light object;<br>-↑ for deceptive vs truthful action;<br><br>FCR CSE: ↑ for heavy object in the deceptive condition (lifted as it was light) vs all conditions.	Muscle specific facilitation during the observation deceptive action, following EMG activation during AE
**Finisguerra et al., 2018**	18 pp (9 male, 24.8 ± 7.55 yr)<br><br>13 pp (5 male)	Reaching, grasping, lifting to place actions performed on a light (100 g) or heavy (550 g) cube by an actor providing:<br>-truthful or<br>-deceptive information about the object weight after being correctly informed about object weight,<br>-or truthful information about the object weight after being deceived about the object weight;<br><br>+EMG recording during AE	Relaxing hand; fixation cross<br><br>Logarithmic transformation and difference from the averaged baselines	FDI<br>ECR	Sp TMS (130% MT)<br>At 1050 ~ 1250 ms from video onset, end of object grasping/first part of the lifting phase.	FDI CSE:<br>-↑ during the observation of deceptive action vs truthful and deceived actions,<br>-↓ during the observation of deceived actions vs truthful and deceptive actions;<br><br>ECR CSE:↑ during the observation of deceptive action vs truthful and deceived actions, without differences between the two genuine conditions.	Dissociable mirroring of high level deceptive intention (muscle independent) and low level kinematic adaptation (muscle specific) of CSE, following EMG activation during AE
**Liuzza et al. 2015**	20 pp (10 male, 24 ±6 yr)	Action with immoral or neutral moral intention (stealing a wallet vs picking up a notepaper, respectively). Participants' personality traits were measured using the temperament and character inventory (TCI).	Black square<br><br>Ratio from baseline	FDI<br>ADM	SpTMS (~120% rMT)<br>At on average 1100ms (±100ms) after the action onset.	FDI-ADM CSE: ↓ when observing immoral actions in individuals with high scores in the harm avoidance TCI subscale.	Moral valence modulation on CSE.

STUDY	METHOD					RESULTS	
	**Sample N (M), age**	**Observed action/ Experimental manipulation**	**Baseline condition /data normalization**	**Muscle (s)**	**Type and time of stimulation**	**CSE Effects during AO**	**Effects**
**Craighero and Mele, 2018**	24 pp (13 male, 21.25 ±1.53 yr)	Loop of hand reaching and squeezing a trumpet, during 3 sessions differing for the positive, negative or neutral effects that the squeezing of the trumpet exerted on a third person. Kinematics and visual context were kept identical while the intention differed.	Fixation cross<br><br>Difference from baseline	OP	SpTMS (~120% MT)<br>At 3000ms after video onset.	OP CSE:<br>-↓ for actions with negative intentions (i.e., unpleasant consequences in the other);<br>-no correlations between MEP values and scores obtained from the IRI and harm avoidance TCI subscale.	CSE.suppression during the observation of actions with negative intentions
**Hogeveen and Obhi 2012**	40 pp (12 male, 18.5 yr) randomly assigned to one of 4 groups:<br><br>-no social interaction/robot observation (10 pp)<br>-no social interaction/human observation (10 pp)<br>-social interaction/robot observation (10 pp),<br>-social interaction/human observation (10 pp).	Videos depicting a human hand or a grabber reaching tool squeezing a ball on a black background.	Fixation cross<br><br>Ratio from baseline	APB	SpTMS (~110-120% rMT).<br>At points of maximum of maximum flexion during squeezes observation on 40% of trials.	APB CSE: ↑ for human actions as compared to robotic actions in participants that were prior engaged in a real social interaction.	CSE modulation after social priming
**Hogeveen et al. 2014**	low-power group:<br>17 pp (4 male, 20.65 ± 2.12 yr)<br><br>high-power group:<br>18 pp (7 male, 20.59 ± 2.12 yr)<br><br>Neutral group:<br>10 pp (2 male, 18.63 ± 0.81 yr)	Hand-squeezing actions after being primed in one out of 3 possible conditions:<br>- low-power: participants wrote an essay about an experience where someone had power over them;<br>- high-power: they wrote about an experience where they had power over someone else;<br>- neutral: they wrote about what happened the day before.	Fixation cross<br><br>Percentage change from baseline	APB	SpTMS (~110-120% rMT).<br>At points of maximum squeeze intensity occurring at 3,128ms, 4,328ms, 5,494ms or 6,728ms after trial onset.	APB CSE:<br>-↓ in the high-power group;<br>-no significant differences between neutral condition and low- and high- power conditions.	Top-down modulation of CSE after priming training of power conditions

STUDY	METHOD					RESULTS	
	Sample N (M), age	Observed action/ Experimental manipulation	Baseline condition /data normalization	Muscle (s)	Type and time of stimulation	CSE Effects during AO	Effects
**Candidi et al., 2014**	9 pianists (7 male, 24–37 yr)<br><br>9 musically naïve students (1 male, 20–33 yr)	Right hand of a professional pianist performing piano scales in correct or incorrect manner.	Static hand<br><br>Difference from baseline	APB<br>FDI<br>ADM	SpTMS (~120% MT).<br>At 100ms, 300ms, or 700ms after video onset.	APB CSE:<br>-↑ somatotopic error-locked facilitation in expert pianists at 300 ms.<br>-no modulation in the musically naïve individuals.	Motor expertise modulation on CSE.
**Aglioti et al., 2008**	10 elite basketball players (23.9 ±3.3 yr)<br><br>5 journalists and 5 coaches (expert watchers)<br>(33.1 ±7.5 yr)<br><br>10 students with no experience playing basketball (25.4 ± 6.8 yr)	Videos showing free basket shots performed by a professional basketball player that could landed in the basket (IN-shots) or not (OUT-shots). Videos were interrupted at different time-points. All videos could last 568ms, 781ms or 1,207ms.	Videos of soccer kicks and static images<br><br>Z-scores	ADM<br>FCU	SpTMS (~130% MT).<br>Between 100 and 400ms before the end of the video presentation.	↑CSE in athletes and watchers during basket but not soccer videos; similar CSE for both sports in novices;<br><br>ADM CSE:<br>-↑ in athletes for OUT than IN shots only when videos lasted 781ms (i.e., when the little-finger angle allowed discriminating between IN and OUT shots in a behavioural task);<br>- no significant differences between shots for novices and expert watchers;	Motor expertise modulation on CSE.
**Amoruso and Urgesi, 2016**	13 pp (5 male, 19–30 yr)	Actions embedded in congruent, incongruent and ambiguous contexts.	Static pictures of squares and circles<br><br>Percentage change from baseline	FDI<br>FCR	SpTMS (~120% rMT).<br>At randomly chosen intervals ranging from 667ms to 967ms after video onset.	FDI CSE: ↓ for actions embedded in incongruent contexts.	Top-down modulations of contextual cues on CSE (suppression triggered by incongruent contexts).
**Amoruso et al., 2016**	15 pp (5 male, 25.86 ± 2.06 yr)	Actions embedded in congruent, incongruent and ambiguous contexts.	Fixation cross<br><br>Percentage change from baseline	FDI<br>FCR	SpTMS (~120% rMT).<br>At 80ms, 240ms or 400ms after video onset.	FDI CSE: ↑ for actions embedded in congruent contexts at ~240ms.<br><br>FDI CSE: ↓ for actions embedded in incongruent contexts at ~400ms.<br><br>No contextual modulations at ~80ms	Top-down modulations of contextual cues on CSE (facilitation triggered by congruent contexts at ~240ms and suppression triggered by incongruent contexts at ~400ms).

STUDY	METHOD					RESULTS	
	**Sample N (M), age**	**Observed action/ Experimental manipulation**	**Baseline condition /data normalization**	**Muscle (s)**	**Type and time of stimulation**	**CSE Effects during AO**	**Effects**
**Riach et al., 2018**	24 pp (16 male, 22.42 ± 3.23 yr)	Action (i.e., pinching a sponge) that could be performed embedded in:<br>- a plain black background;<br>- a background containing objects that were incongruent with the observed activity;<br>-a background containing objects that were congruent with the activity.	Static hand and plain black background<br><br>Z-scores	FDI<br>ADM	SpTMS (~110% MT).<br>At 1700ms or 4900ms after video onset, at the point of maximal contraction of the FDI muscle during sponge pinch.	FDI CSE: ↑ for actions embedded in congruent contexts.	Top-down modulations of contextual cues on CSE.
**Cretu et al. 2019**	EXP1: 24 pp (7 male, 24.5 ± 4.73 yr)<br><br>EXP2: 29 pp (13 male, 23.5 ± 2.66 yr)	Full or occluded video-clips of an actor grasping and lifting a jar using a precision vs. whole-hand grips:<br>-Grip types were associated to color cues that could reliable predict the upcoming grasp action (i.e., blue square for WHG; white for PG);<br>-Half of the videos were preceded by informative cues, while the other half were preceded by an uninformative cue (grey square) that could be followed by either of the two grasps;<br>+EMG recording during AE.	Black screen<br><br>Difference from baseline	FDI<br>ADM	SpTMS (130%rMT).<br>During:<br>-reaching (~2 sec after video onset),<br>-grasping (~3.48 after video onset),<br>-during lifting (~4.92 sec after video initiation).	EXP1<br>FDI and ADM CSE: grip-specific modulation during the observation of both full and occluded actions<br><br>EXP2<br>FDI and ADM CSE:<br>-grip-specific modulation during the observation of both full and occluded actions if preceded by informative cues;<br>-grip-specific modulation during the observation of full actions only.	Motor representations activated by contextual cues even in the absence of kinematic stimuli, following EMG activation during AE<br><br>Top-down modulations of contextual cues on CSE.

STUDY	METHOD					RESULTS	
	Sample<br>N (M), age	Observed action/<br>Experimental manipulation	Baseline condition /data normalization	Muscle (s)	Type and time of stimulation	CSE Effects during AO	Effects
Janssen, Steenbergen & Carson, 2013	13 pp (6 males, 18–38 yr)	Reaching-and-grasping actions upon an object composed of a black bar and a white disk. The object could be inserted into a small hole through a WHG of the white disk or into a large hole, through a PG of the black bar.<br>An arrow cues between the two holes could point either to the small or to the large hole, indicating the upcoming goal. Actions could be preceded by congruent (67% of trials) or incongruent (22%) arrow cues.<br><br>+EMG recording during AE.	Eyes closed	FDI<br>ADM	SpTMS (120% rMT).<br>At 3,000ms, 4900ms, 5280ms, 5680ms, 6080ms, and 7280ms after trial onset.	ADM and FDI CSE: ↑ during the observation of actions preceded by a congruent cue, in accordance with the muscles involved in the observed action.<br>FDI CSE: ↓ for incongruent cues (i.e., consistent with the action specified by the cue, rather than the observed one).	Top-down modulation of symbolic cues on CSE.
Mattiassi et al., 2014	22 pp (11 male, 23 ± 4 yr)	Static images of either still or moving hands (i.e., index finger and little finger movements) preceded by masked primes of the same or different movements.	Blank screen<br><br>Percentage change from baseline	FDI<br>ADM	SpTMS (120% rMT).<br>At one of two moments during the trial, either 133ms (early delay) or 307ms (late delay) after the onset of the probe.	FDI and ADM CSE: ↑ for action probe (consciously perceived) as compared to baseline (i.e., still hand).<br><br>FDI and ADM CSE: ↓ at ~307ms after probe onset for movements preceded by incongruent masked primes.	CSE suppression for unconscious presentation of incongruent actions

STUDY	METHOD					RESULTS	
	Sample N (M), age	Observed action/ Experimental manipulation	Baseline condition /data normalization	Muscle (s)	Type and time of stimulation	CSE Effects during AO	Effects
**Sartori et al., 2013**	30 pp (8 male, 21 ± 5 yr)	Pouring sugar action with a spoon performed by using a precision grip (PG) or pouring coffee action with a thermos using a whole-hand grip (WHG) into different coffee cups/mugs. Critically, the 3 first cups/mugs where located on the table near the model performing the actions, while the fourth one was located far away, near the observer. Anyone on that side of the table wanting to pick up the cup/mug would need to use a PG/WHG.	Fixation cross<br><br>Ratio from baseline	FDI<br>ADM	SpTMS (~110% MT).<br>At five time points: T1, first contact with the object (i.e., spoon or thermos) around 1550ms; T2, when pouring sugar/coffee into the third cup (~6530ms); T3, when the hand get away from the cup (~6680ms); T4, when model's wrist trajectory move towards the 4th cup in the social condition (or not, in the non-social one, ~6950ms); T5, the model's arm reaches the 4th cup (or not, ~7160ms).	ADM CSE: ↑ for the social than for the non-social condition at T4 and T5 (i.e., timing of the complementary request) when the request demanded a WHG and ↓ when the request demanded a PG.<br><br>Grasp modulation<br>ADM CSE: ↓ at T1, T2, and T3 for actions performed with PG (i.e., grasping the spoon) indicating emulation (mimicking AO and ignoring the action goal).<br>↑ at T4 and T5 for actions requesting a WHG (i.e., model pouring coffee in a mug), indicating motor resonance in terms of reciprocity.	Social context modulation (i.e., shift from emulation to reciprocity) on CSE.

Table 1. List of the revised studies. Method of the studies, sample features, observed action, recorded muscles, stimulation protocols and timing of stimulation, results and interpretation of the effects, are shown. Only the effects on CSE as measured via MEP during AO are considered.

↑ increased; ↓ decreased; ADM Abductor Digiti Minimi; AE Action Execution; AH Abductor Hallucis; AO Action Observation; APB Abductor Pollicis Brevis; ECR Extensor Carpi Radialis; EDC Extensor Digitorum Communis; EIP Extensor Indicis Proprius; EMG Electromyography; FCR Flexor Carpi Radialis; FCU Flexor Carpi Ulnaris; FDI First Dorsal Interosseus; FDS Flexor Digitorum Superficialis; MT Motor Threshold; NA Not available; OA Observed Action; OP Opponens Pollicis; PG Precision grip; Pp Participants; QF Quadriceps Femori; SI Stimulation Intensity; Sp Single pulse; WHG Whole Hand Grip; Yr years
